# A compact fiber‐optic probe‐based singlet oxygen luminescence detection system

**DOI:** 10.1002/jbio.201600078

**Published:** 2016-07-25

**Authors:** Nathan R. Gemmell, Aongus McCarthy, Michele M. Kim, Israel Veilleux, Timothy C. Zhu, Gerald S. Buller, Brian C. Wilson, Robert H. Hadfield

**Affiliations:** ^1^ Division of Electronic and Nanoscale Engineering University of Glasgow UK; ^2^ Institute of Photonics and Quantum Sciences Heriot‐Watt University Edinburgh EH14 4AS UK; ^3^ Department of Radiation Oncology University of Pennsylvania USA; ^4^ University Health Network/University of Toronto Canada

**Keywords:** singlet oxygen, fluorescence, quantum optics, photon counting

## Abstract

This paper presents a novel compact fiberoptic based singlet oxygen near‐infrared luminescence probe coupled to an InGaAs/InP single photon avalanche diode (SPAD) detector. Patterned time gating of the single‐photon detector is used to limit unwanted dark counts and eliminate the strong photosensitizer luminescence background. Singlet oxygen luminescence detection at 1270 nm is confirmed through spectral filtering and lifetime fitting for Rose Bengal in water, and Photofrin in methanol as model photosensitizers. The overall performance, measured by the signal‐to‐noise ratio, improves by a factor of 50 over a previous system that used a fiberoptic‐coupled superconducting nanowire single‐photon detector. The effect of adding light scattering to the photosensitizer is also examined as a first step towards applications in tissue *in vivo*.

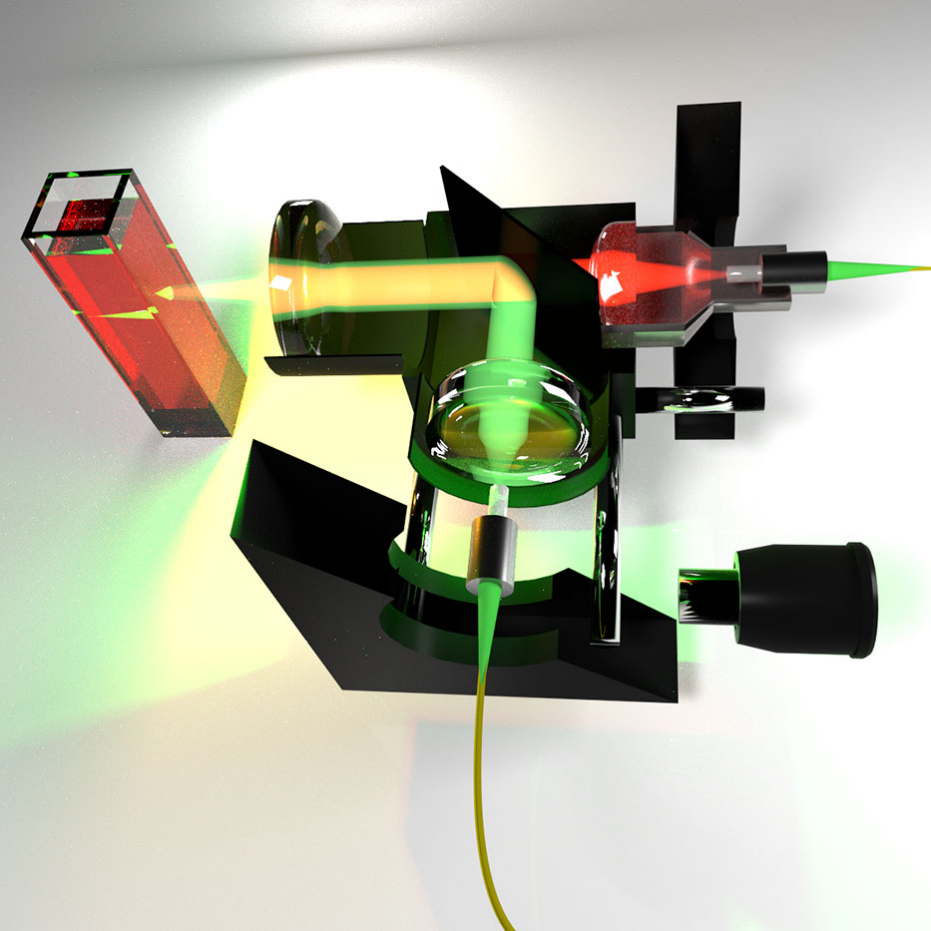

## 1 Introduction

**Figure 1:**
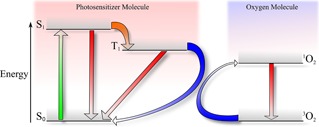
Simplified Jablonski diagram showing generation of singlet oxygen (^1^O_2_) from a type II photosensitizer. Photon absorption excites the photosensitizer from its ground state, *S*_0_, to an excited singlet state, *S*_1_. Decay of this excited state can occur via fluorescence back to the ground state or by intersystem crossing to a triplet state, *T*_1_. The *T*_1_ → *S*_0_ transition by phosphorescence emission is quantum mechanically forbidden, so that *T*_1_ is long lived, allowing energy transfer to triplet ground‐state molecular oxygen, ^3^O_2_, generating ^1^O_2_. A 1270 nm photon is emitted on decay of ^1^O_2_ to ^3^O_2_.

Singlet oxygen (^1^O_2_), the first electronic excited state of the oxygen molecule, is involved in many biological processes 1, 2, including serving as a major cytotoxic photoproduct in photodynamic therapy (PDT) for treatment of cancer, vascular pathologies, skin conditions and localized infection 3. PDT uses light‐activated photosensitizing compounds to cause cell death and offers a potential locally‐curative treatment. However, PDT is an inherently complex process in which the photosensitizer (PS), light and molecular oxygen vary inter‐dependently and dynamically during treatment 4. For example, the effective absorption of treatment light is affected by the local concentration of PS, which in turn is affected by the blood concentration and oxygenation of the tissue. The oxygen distribution may vary during treatment due to photodynamic consumption and PDT‐induced changes in blood flow 5. The effective PS concentration and distribution may also change during treatment due to ^1^O_2_‐mediated photo‐bleaching. These combined effects make reliable PDT dosimetry extremely challenging, but this is important to achieve optimal PDT efficacy and safety, particularly when there is curative intent 6. ^1^O_2_ is generated in Type‐II photosensitizers as shown in Figure 1 7. Direct measurement of ^1^O_2_ constitutes the “gold standard” of PDT dosimetry 8, against which other indirect techniques, such as measuring the PS fluorescence photobleaching 9 or modelling the entire photophysical process 10, can be compared 11. However, direct detection of this excited state is extremely challenging, since it relies on detecting the 1270 nm emission at from the ^1^O_2_ → ^3^O_2_ transition 12: in biological media this has low probability (∼10^–7^) and short lifetime (≪1 µs) due to the high reactivity of singlet oxygen with biomolecules. In addition, most off‐the‐shelf photodetectors have low sensitivity in this spectral region.

Generation of ^1^O_2_ requires the presence of a photosensitizer, pump illumination and oxygen. For short‐pulse illumination, the temporal profile is described by Eq. (1) 7, 11:

(1) $${[}^{1}O_{2}](t)=N\sigma [S_{0}]\;\Phi_{D}\frac{\tau_{D}}{\tau_{T}-\tau_{D}}\left(\text{exp}\left(\frac{-t}{\tau_{T}}\right)-\text{exp}\left(\frac{-t}{\tau_{D}}\right)\right)$$

where [^1^O_2_] is the singlet oxygen concentration at time,* t*, after an illumination pulse, *N* is the number of photons in the illumination pulse, *σ* is the PS absorption cross‐section, *S*_0_ is the photosensitizer concentration, *Φ*_D_ is the singlet oxygen quantum yield, *τ*_T_ is the photosensitizer triplet‐state lifetime and *τ*_D_ is the singlet oxygen lifetime. *τ*_T_ and *τ*_D_ depend strongly on the local microenvironment 7, 13.

Until recently, the most detailed studies of singlet oxygen luminescence dosimetry (SOLD) used extended‐wavelength photomultiplier tubes as the best‐available detector, although the quantum efficiency was typically <∼1% thus requiring bulky optics to improve collection efficiency 14, 15, 16. Recently, however, both superconducting nanowire single‐photon detectors (SNSPDs) 17 and semiconductor‐based single‐photon avalanche diodes (SPADs) 18 have become available that promise substantially higher quantum efficiency and more flexible implementation. We previously reported successful ^1^O_2_ luminescence detection in PS solution using an SNSPD detector coupled to a single‐mode optical fiber 19. Similar results were recently presented using a free‐running InGaAs/InP SPAD, however while the detector itself was fiber coupled, the collection optics remain bulky and impractical for a clinical setting 18. Here, we present analogous studies utilizing a gated InGaAs/InP SPAD detector. Such gated SPADs do not require the same cooling restrictions as free‐running SPADs, using compact Peltier cooling instead of the bulky Stirling systems of 18, and as such they are much more portable, reliable, and practical. By synchronizing the detector gating with the frequency of the pump laser source, we were able to create a gating pattern that allows the detector to be active only during important time windows (i.e. when the ^1^O_2_ luminescence signal is expected), thereby reducing the large background signal due to the relatively prompt PS luminescence. The system was further enhanced by the use of a remote sensor head in which the pump laser and luminescence signal are coupled into optical fibers, permitting the detection and timing electronics to be housed remotely from the treatment site. This innovation is an important step forward in the development of a practical SOLD system for eventual clinical use.

## 2 Experimental setup

**Figure 2:**
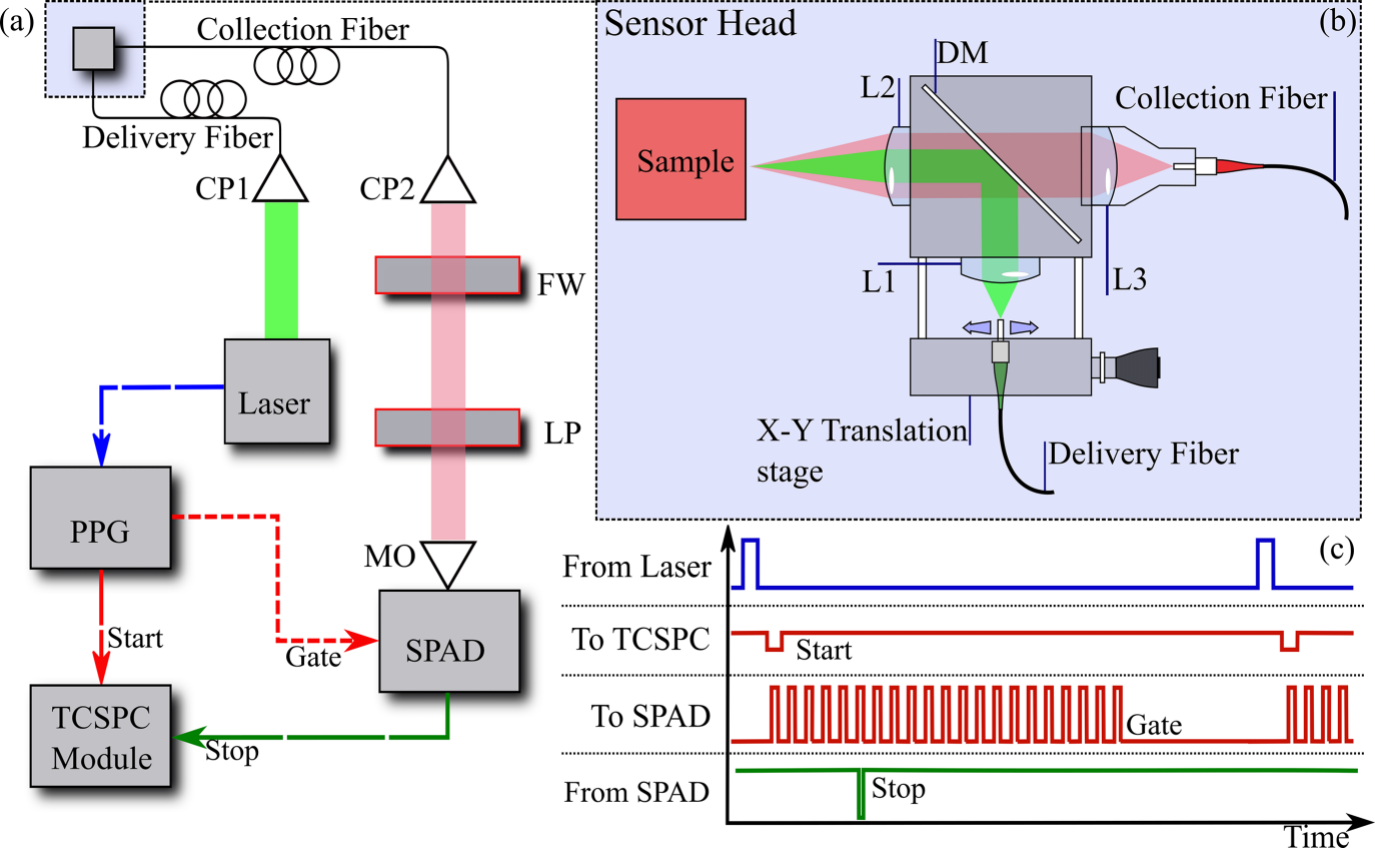
(**a**) Block diagram of the experimental arrangement using the SPAD. The 523 nm laser is coupled into the delivery fiber via a collimation package (CP1) in the remote sensor head. Light from the collection fiber is coupled out through CP2, directed through a filter wheel (FW) for the band‐pass filter selection, then through a long pass filter (>1000 nm). A microscope objective (MO) images the fiber core onto the face of the detector. (**b**) A schematic of the sensor head showing the lenses (L1–L3) and dichroic mirror (DM). The *x*–*y* translation stage allows the two optical axes to be aligned on the sample. (**c**) An electrical signal (blue) to the PPG triggers the release of two signals (red). One acts as the TCSPC ’start' signal, while the other triggers the SPAD gating. The output from the SPAD (green) – which can only occur within a gated period – is sent to the TCSPC stop ’channel'.

**Figure 3:**
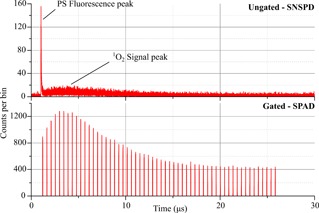
Example histograms of the 1270 nm ^1^O_2_ luminescence signal from Rose Bengal in water from a free‐running detection system (top, from 19), and the gated system presented here (bottom). Both histograms have the same bin width (1.024 ns) and acquisition time (10 minutes). The position of the histogram along the *x*‐axis may differ due to different optical and electrical signal path lengths.

A block diagram of the experimental setup is shown in Figure 2(a). The pump illumination is provided by a Q‐switched frequency‐doubled Nd : YLF laser (CrystaLaser, QL‐523‐200‐S) that emits 10 ns duration, 523 nm wavelength pulses at a repetition rate of 18.2 kHz with an average power of 200 mW. The detector used was a Peltier‐cooled InGaAs/InP single photon avalanche diode (SPAD) detector (Model: IR‐DH‐025‐C, Micro Photonic Devices, Italy) with ∼30% detection efficiency at a wavelength of 1270 nm, operating at a temperature of ∼230 K. The SPAD active region was 25 µm in diameter. The detector operation and gating mechanism have been described elsewhere 20 and here we will describe only the method by which a patterned gate is applied to the counts *vs*. time histogram. As shown in Figure 2(c), the laser outputs a synchronous electrical signal that is sent to a programmable pulse pattern generator (PPG: Agilent 81110A). Each pulse generates outputs on two separate channels, each with a pulse shape designed to match the intended input. The first output is a single pulse sent to the sync or ’start' channel of the time‐correlated single‐photon counter (TCSPC, PicoQuant HydraHarp), while the second is a pattern of pulses (at a higher frequency than the laser repetition rate) sent to the SPAD control module. Whenever the latter receives a pulse from the PPG, the SPAD is turned on for a pre‐determined duration, referred to here as the gate‐width (typically much shorter than the laser period, ∼24 ns). Any detection events (or dark counts) falling within this gate‐width trigger the output of a pulse that is sent to the ’stop' channel of the TCSPC. Any photons incident on the detector outside of the gate‐width cannot be detected. This approach enables construction of a timing histogram in which detection events are generated only within the pre‐selected gate windows; thus, we can intentionally avoid the detection of photons at times where few photons are expected (for example, towards the end of the histogram) or where unwanted fluorescence photons will saturate the detector (at the start of the histogram). This strategy also reduces the overall count rate of the detector, helping to avoid the effects of pulse pile‐up from distorting the shape of the histogram, and reducing the effects of after‐pulsing which leads to increased background levels. This approach leads to an inevitable loss of signal photons being collected at times outside the detector gating periods. However, this loss of signal appears to have little effect of the quality of data shown in Figure 3, which shows the difference in histograms collected with a free running detector (data is from the SNSPD setup from 19) and those collected with the setup used here.

A schematic of the portable compact sensor head is shown in Figure 2(b). Pump laser light is collimated from the 200 µm core diameter optical fiber by a 35 mm focal length achromatic lens (L1). A dichroic mirror (DM, Thorlabs) reflects all light <950 nm and hence reflects the pump laser to an uncoated plano‐convex 75 mm focal length lens (L2) to create a ∼430 µm diameter illumination spot at the sample. The average optical power at the sample is ∼30 mW. L2 collects the resulting emission from the sample, comprising the ^1^O_2_ luminescence and background emission. By using the transmission port of dichroic beamsplitter for the collected emission, the majority of the shorter wavelength PS fluorescence is removed, before a 37 mm fixed‐focus collimator (L3) couples the remaining emission into a 105 µm diameter core fiber. The de‐magnification provided by the lens pairing of L2 and L3 corresponds to a detection window of ∼212 µm in diameter (0.0353 mm^2^ area) at the sample surface. An *x*–*y* translation stage allows the delivery fiber to be moved, ensuring overlap of the detection and excitation channels within the sample. Light is coupled out of the collection fiber through an 18 mm focal length collimator (CP2) and is directed through a filter wheel (FW) and then a long‐pass filter (Thorlabs FELH1000) with 95% transmission >1000 nm and in‐band transmission of ∼10^–4^%. The filter wheel allows selection of one of 5 band‐pass filters with central wavelengths at 1210 nm, 1240 nm, 1270 nm, 1310 nm, and 1340 nm. Each filter has a spectral full‐width at half maximum (FWHM) of 20 nm and a maximum transmission of ∼60% at the central wavelength. These filters sample the spectrum of detected light so that the residual background due to PS luminescence can be subtracted from the true ^1^O_2_ signal. A long working distance microscope objective (8.5 mm focal length) focuses the emission onto the sensitive area of the detector, so that the 105 µm diameter fiber core is imaged to a ∼50 µm spot at the detector surface, which has an active area of only 25 µm diameter. Clearly, over‐filling of the detector active area results in loss of efficiency (∼25%), however the mismatch in illumination and detector areas ensures a degree of overlap that minimizes loss of signal due to any alignment drift. The larger collection fiber core also provides a higher system numerical aperture (NA = 0.22), further improving the overall system throughput. Approximately 11% of the 1270 nm photons collected by the objective lens reach the detector, which has a single‐photon detection efficiency of ∼30%.

## 3 Singlet oxygen detection in photosensitizer solution

**Figure 4:**
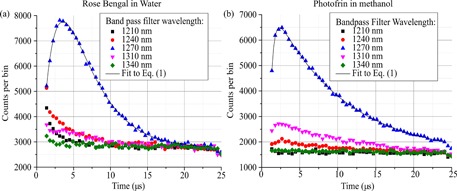
(**a**) Histograms acquired with each band‐pass filter using 300 s integration for a 50 µg/ml sample of Rose Bengal in water. (**b**) Corresponding histograms acquired over 120 s for a 50 µg/ml sample of Photofrin in methanol. The solid curves show the best fit to Eq. (1).

Measurements were made in two different model photosensitizers: Rose Bengal in water and Photofrin in methanol. Rose Bengal is a well‐characterized single molecular compound and allows direct comparison with our previous SNSPD measurements 19, while Photofrin is an established first‐generation clinical photosensitizer that was dissolved in methanol to ensure that it was fully disaggregated and hence photodynamically active. Figure 4 shows histograms taken for each of the bandpass filters for each photosensitizer. The corresponding triplet‐state and singlet oxygen lifetimes calculated by fitting the histograms (1270 nm minus the background taken at 1210 nm) to Eq. (1) are shown in Table 1, together with published values (similarly calculated from fits to time correlated single photon counting histograms): as mentioned above, the solvent and photosensitizer can significantly alter the transition reaction rates. A constant offset was added to the fitting of Eq. (1) as a free parameter to account for a shift in background counts between the subtracted histograms. The errors quoted are the standard error for the fitting that was performed by Origin software with a Levenberg–Marquardt algorithm to iterate the parameter values.

**Table 1:** Measured lifetimes (in µs) from fits to Eq. (1) for Rose Bengal in water and Photofrin in methanol, together with corresponding literature values

	Rose Bengal in water	Published values	Photofrin in methanol	Published values
PS triplet state lifetime, *τ_T_*	2.1 ± 0.2	2.3 ± 0.3^a^	0.43 ± 0.03	0.3 ± 0.2^b^
Singlet oxygen lifetime, *τ_D_*	3.8 ± 0.3	3.0 ± 0.3^a^	9.4 ± 0.2	9.0 ± 0.5^b^

Published values from Refs:

a 25

b 19

For each photosensitizer the 1270 nm wavelength signal in Figure 4 stands clearly above the counts at the wavelengths on either side, confirming that the signal was predominantly from ^1^O_2_ luminescence. The integration time with Rose Bengal was ∼2.5 times that for Photofrin in methanol, for approximately the same counts after background subtraction. The increased signal from Photofrin is due to a longer singlet oxygen lifetime coupled with a shorter PS triplet‐state lifetime causes an overall increase in the probability of decay via photon emission. Using the 1310 nm filter it is still possible to see a small fraction of the ^1^O_2_ signal from Photofrin due to the non‐symmetrical spectral response of the filters causing a small spectral overlap between the ^1^O_2_ luminescence and the 1310 nm wavelength filter bandwidth.

As a comparison to our previous ^1^O_2_ luminescence measurements using an SNSPD detector, Table 2 shows the 1270 nm count rates for three systems: the SPAD fiber probe system presented here, and both free‐space‐coupled and fiber‐coupled SNSPD systems reported in 19. The collection rate for the SPAD system is much higher than that of the other two systems, which translates directly into shorter histogram acquisition times as required to be clinically practical. While the SNSPD free‐space system displays a better signal‐to‐background ratio (SBR), this comes at the expense of the overall photon collection efficiency, which is limited by the single‐mode fiber coupling to the very small SNSPD (9 µm diameter). Thus, SBR is not a sufficient measure of the overall efficacy. Rather, we define the signal to noise ratio (SNR) as:

(2) $$\text{SNR}=\frac{N_{s}}{\sqrt{N_{s}+N_{b}}}$$

where *N*_s_ and *N*_b_ are the signal and background count rates, respectively. This provides information on the quality of the signal. Thus, the SNRs for the three systems are also shown in Table 2. The improvement in the SPAD over the SNSPD is due mainly to the larger sensor area (25 µm versus 9 µm diameter) that enables use of a larger‐core multimode collection fiber, which also improves the NA of the collection optics.

**Table 2:** Collection and background count rates for ^1^O_2_ luminescence detection from 50 µg/ml Rose Bengal in water for the different detection schemes

	Signal count rate (cps)	Background count rate (cps)	SNR
SNSPD, free space	∼65	∼60	5.8
SNSPD, fiber coupled	∼0.6	∼10	0.2
SPAD, fiber coupled	∼295	∼515	10.4

## 4 Measurements in the presence of scattering

**Figure 5:**
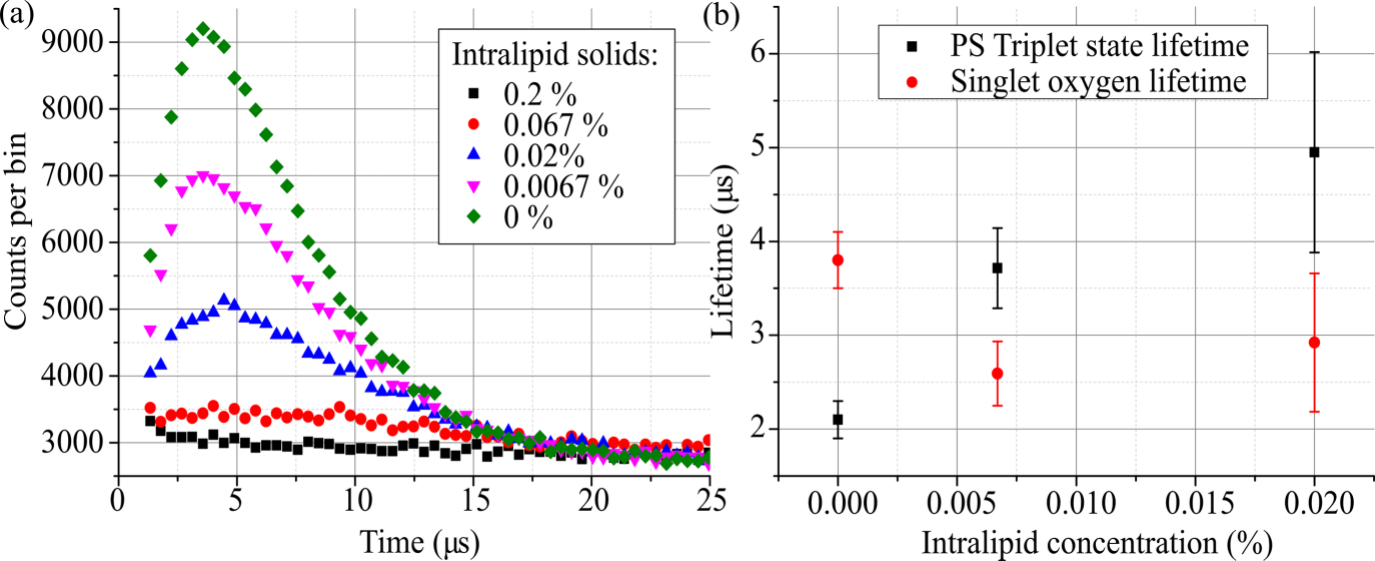
(**a**) Histograms acquired for 300 s integration from a 50 µg/ml sample of Rose Bengal in water with increasing Intralipid concentration. (**b**) Lifetimes of ^1^O_2_ and PS triplet state: error bars represent the standard error in the fit to Eq. (1).

A lipoprotein suspension (Intralipid), which is commonly used to simulate tissue light scattering 21, was added in increasing concentration to a cuvette containing 50 µg/ml Rose Bengal. The resulting histograms [Figure 5(a)] show the decrease in ^1^O_2_ luminescence counts due to either i) diffusion of the excitation and 1270 nm light into a larger volume, such that a smaller fraction is captured by the collection optics, and/or ii) quenching of the singlet oxygen signal via the additional de‐excitation pathways as a result of interaction with the Intralipid solids, and/or iii) altered *τ*_T_ and *τ*_D_, as evidenced in Figure 5(b). The lengthening of *τ*_T_ and reduction of *τ*_D_ reduce the probability of photon emission and also spread the signal across a greater number of time bins. These altered lifetimes have been reported previously when protein is added to photosensitizer solutions 14, 19 and is also expected *in vivo* due to proteins and other biomolecules. The Intralipid concentration of 2% most closely represents the light scattering of tissue 22, so that the rapid loss of signal seen in Figure 5, even in the absence of absorption other than that of the photosensitizer itself, suggests that the signal will be very weak in the real *in vivo* situation. On the other hand, successful detection of singlet oxygen luminescence has been reported in tissues *in vivo* using extended‐red photomultiplier tubes. Although these have significantly lower quantum yield at 1270 nm (<1% compared with ∼30% for the SPAD used here), they have much larger effective detection area (typically ∼20 mm^2^ compared ∼5 × 10^–4^ mm^2^). Hence, there is a need to further reduce the SPAD system noise (dark counts and background photons) and increase the detector efficiency and optical system throughput. The use of large area or arrayed InGaAs/InP SPADs detectors 22 will also be an important consideration for future implementations of this approach. While arrays of InGaAs diodes has been used in Singlet Oxygen imaging 23, 24, the sensitivity, cost, and optics involved, would make them difficult to implement in a clinical dosimetry system.

## 5 Conclusions

The system presented here represents an advance in SOLD through the development of a compact singlet oxygen sensor head and a user‐friendly detection system for direct PDT dosimetry that could be used both for preclinical PDT research in animal models* in vivo* and in clinical trials. The fiber‐optic coupled sensor head allows easier positioning onto the target tissue (e.g. tumor) than the fixed and bulky optical systems used previously, while the SPAD detector does not require the separate bulky cryogenic unit used with SNSPDs. The gated operation of the SPAD significantly reduces the probability that the detector is ‘blinded' by the strong and prompt fluorescence/phosphorescence signal, while simultaneously reducing the dark counts. These are key factors in the operation of a detector with a long recovery time used for singlet oxygen luminescence detection systems. The system has been tested with known photosensitizers and proved capable of determining lifetimes consistent with those obtained using bulkier and less portable systems. The overall performance in terms of count rate and signal‐to‐noise is improved by ∼50‐fold over the fiber‐coupled SNSPD system. The next stage in development and testing will be to carry out the first* in vivo* studies using this fiber based and user friendly system.

## Supporting information

Author biographies
