# Supplementary material for: A compact fiber‐optic probe‐based singlet oxygen luminescence detection system
Source: J Biophotonics. 2016 Jul 25;10(2):320–6. doi: 10.1002/jbio.201600078 (PMC5266677; doi:10.1002/jbio.201600078)
Supplement: Supplementary file 1 — Author biographies [file JBIO-10-320-s001.pdf]

Author Biographies

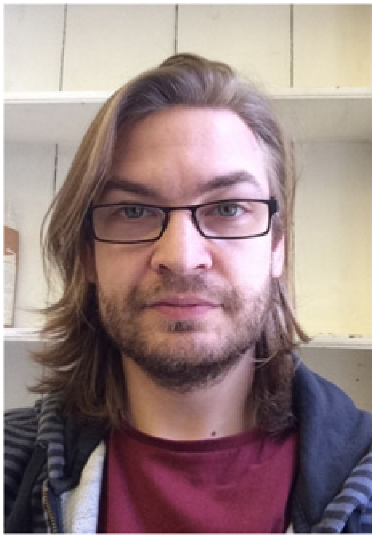

Nathan Gemmell received a BSc in Physics with Nuclear Astrophysics and MSc in Radiation Detection and Instrumentation from the University of Surrey. He completed his PhD in Infrared Single-Photon Sensing from Heriot-Watt University in 2015. He is currently working as a Research Assistant at Glasgow University as part of the quantum technologies hub, QuantIC. Dr Gemmell's research interests focus on applications of the time resolved detection of single infrared photons.

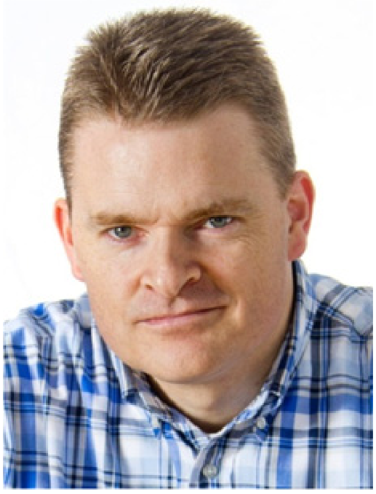

Aongus McCarthy received the B.Sc. degree from University College Galway, Ireland, in 1989, the Diploma degree in electronic engineering from the Institute of Technology, Carlow, Ireland, in 1990, and the B.Sc. degree in physical optoelectronics from Essex University, Essex, U.K., in 1991. He then worked in industry before completing the Ph.D. degree in physics from Heriot-Watt University, Edinburgh, U.K., in 2002. He is currently a Research Fellow with the School of Engineering and Physical Sciences, Heriot-Watt University. His research interests include optical and optomechanical system design, time of-flight depth imaging, single-photon counting technologies, and microscope systems. Dr. McCarthy is a member of the Optical Society of America and the IEEE Photonics Society.

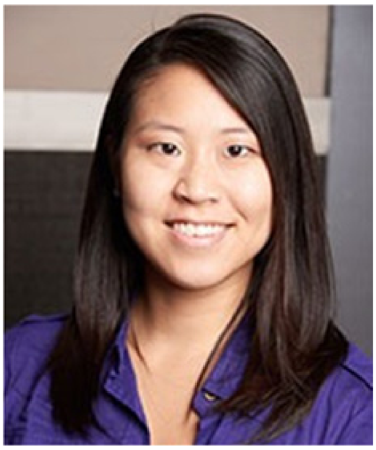

Michele M. Kim received her BA and MS degrees in physics and biophysics from the University of Pennsylvania in 2012 and is currently a PhD candidate at the University of Pennsylvania in physics while pursuing a certificate in medical physics. Her research topics include preclinical and clinical photodynamic therapy dosimetry.

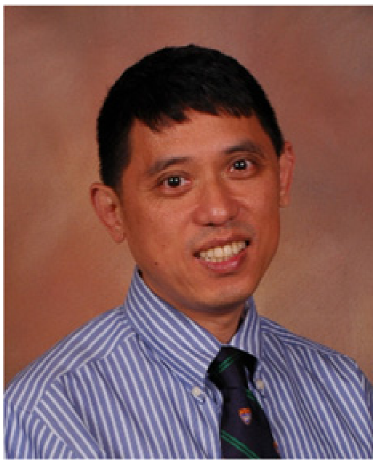

Timothy C. Zhu received his Ph.D in 1992 in Physics from Brown University. He is currently a professor at the Radiation Oncology department of the University of Pennsylvania. His current research interests include explicit PDT dosimetry, Singlet oxygen (SO) dosimetry, integrated system for interstitial PDT, diffuse optical tomography, *in-vivo* dosimetry, and external beam radiation transport.

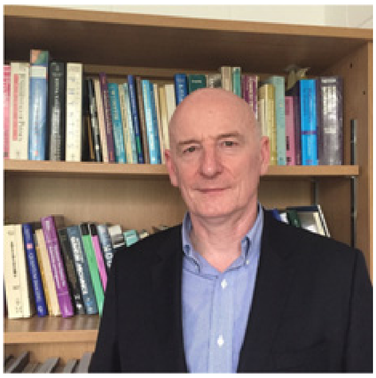

Gerald Buller holds a BSc (Hons) degree in Natural Philosophy from the University of Glasgow and a PhD from Heriot-Watt University, Edinburgh. He is a Professor of Physics at Heriot-Watt University and an EPSRC Established Career Fellow in Quantum Technology. His research interests are single-photon physics and technology, including sparse photon depth imaging, next generation single-photon detectors, and quantum communications.

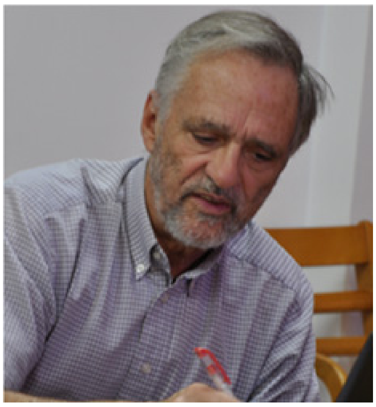

Brian Wilson holds a PhD in Natural Philosophy from Glasgow University, Scotland. This was followed by faculty positions in medical research institutes/hospitals in England, Australia and Canada. Since 1993 he has been Professor of Medical Biophysics in the Faculty of Medicine and Princess Margaret Cancer Centre, University of Toronto, where he directs a biophotonics research program, with emphasis on clinical translation.

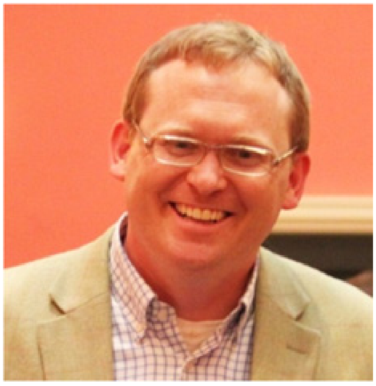

Robert Hadfield is Professor of Photonics in the School of Engineering at the University of Glasgow UK. He received his PhD from the University of Cambridge, UK in 2003. He carried out postdoctoral research at the US National Institute of Standards and Technology, before returning to the UK as a Royal Society University Research Fellow in 2007. He currently holds a European Research Council Consolidator Grant. His research interests centre on advanced infrared photon counting technologies and applications. He has published over 70 peer-reviewed papers and several influential topical reviews in this field.
